# Effects of exercise induced muscle damage on cardiovascular responses to isometric muscle contractions and post-exercise circulatory occlusion

**DOI:** 10.1007/s00421-023-05255-8

**Published:** 2023-06-27

**Authors:** Fabio Zambolin, Tiago Peçanha, Susan Pinner, Massimo Venturelli, Jamie Stewart McPhee

**Affiliations:** 1 Department of Sport and Exercise Sciences, Manchester Metropolitan University, Manchester, UK; 2 Manchester Metropolitan University Institute of Sport, Manchester Metropolitan University, Manchester, UK; 3 Department of Neurosciences, Biomedicine and Movement Sciences, University of Verona, Verona, Italy; 4 Department of Internal Medicine, University of Utah, Salt Lake City, USA

**Keywords:** Exercise-induced muscle damage, Metaboreflex, Exercise pressor reflex, Pain, Soreness, Group III-IV muscle afferents

## Abstract

**Purpose:**

The aim of the present study was to investigate whether exercise-induced muscle damage (EIMD) influences cardiovascular responses to isometric exercise and post-exercise circulatory occlusion (PECO). We hypothesized that EIMD would increase muscle afferent sensitivity and, accordingly, increase blood pressure responses to exercise and PECO.

**Methods:**

Eleven male and nine female participants performed unilateral isometric knee extension at 30% of maximal voluntary contraction (MVC) for 3-min. A thigh cuff was rapidly inflated to 250 mmHg for two min PECO, followed by 3 min recovery. Heart rate and blood pressure were monitored beat-by-beat, with stroke volume and cardiac output estimated from the Modelflow algorithm. Measurements were taken before and 48 h after completing eccentric knee-extension contractions to induce muscle damage (EIMD).

**Results:**

EIMD caused 21% decrease in MVC (baseline: 634.6 ± 229.3 N, 48 h: 504.0 ± 160 N), and a 17-fold increase in perceived soreness using a visual-analogue scale (0–100 mm; VAS_SQ_) (both p < 0.001). CV responses to exercise and PECO were not different between pre and post EIMD. However, mean arterial pressure (MAP) was higher during the recovery phase after EIMD (p < 0.05). Significant associations were found between increases in MAP during exercise and VAS_SQ_, Rate of Perceived Exertion (RPE) and Pain after EIMD only (all p < 0.05).

**Conclusion:**

The MAP correlations with muscle soreness, RPE and Pain during contractions of damaged muscles suggests that higher afferent activity was associated with higher MAP responses to exercise.

## Introduction

Skeletal muscle myelinated type-III (Aδ) and unmyelinated type-IV (C-fiber) nerve afferents detect mechanical and metabolic stimuli (Kaufman et al. 1983; Kaufman and Hayes 2002; Alam and Smirk 1937). Their activity is proportional to contractile tension and the rate of metabolites accumulation, and they modulate perceptions of effort, fatigue, and pain (Pollak et al. 2014). These sensory neurons also orchestrate the afferent arch of the exercise pressor reflex (EPR) (McCloskey and Mitchell 1972) to influence respiratory, cardiac and vascular responses to exercise (Goodwin et al. 1972). However, their typical function can be altered in disease states (Vianna and Fisher 2019) and also by inflammation and pain incurred with exercise-induced muscle damage (EIMD) (Zambolin et al. 2022; Murase et al. 2010; Mizumura and Taguchi 2016).

EIMD is particularly prevalent after unaccustomed eccentric contractions and is characterised by muscle weakness and soreness lasting several days. The soreness occurs due to an increased sensitisation of mechano and nociceptive muscle afferents, causing mechanical hyperalgesia (Kindig et al. 2007; Fujii et al. 2008; Ota et al. 2013; Matsubara et al. 2019; Murase et al. 2021, 2010). This, in turn, is associated with localised inflammation (Armstrong 1984), and increased abundance of metabolites (Ota et al. 2013; Matsubara et al. 2019; Murase et al. 2010). It is speculated that EIMD-induced increase in metabolites might stimulate the metabosensitive muscle afferents and exacerbate cardiovascular responses to exercise. Indeed, increased concentration of ATP, Lactate and H^+^ and deprotonated phosphate (Pi) have been shown to increase metabosensensitive afferent activation producing an increase in the pressor response to exercise (Sinoway et al. 1994; MacLean et al. 2000) and contributing to sensations of pain and fatigue (Jankowski et al. 2013; Pollak et al. 2014). Therefore, it is expected that the change of the inflammatory environment following EIMD (Sandkühler 2009; Queme et al. 2013; Mizumura and Taguchi 2016) may in turn sensitise metabosensitive afferents resulting in an increased cardiovascular and pressor response to muscle contractions. Furthermore, studies of rat muscle suggest that lower pH during contractions may sensitise metaboreceptors to mechanical stimulus (Hotta et al. 2010). However, previous studies into the effect of EIMD on cardiovascular responses to exercise reported contrasting results. Miles et al.(1997) observed increased blood pressure and heart rate responses following EIMD of arm muscles, while Ray et al. (1998) reported no change to blood pressure responses following EIMD compared with pre-EIMD conditions (Ray et al. 1998; Miles et al. 1997). However, methodological differences between these studies, such as differences in relative and absolute force values, muscle group, and exercise intensity make it difficult to understand the physiological mechanisms underpinning these responses. Moreover, most of these previous studies have investigated the effects of EIMD on cardiovascular responses to exercise, which are known to be regulated not only by the muscle afferents but also by the central command and arterial baroreflex (Murphy et al. 2011). A methodology commonly applied to distinguish between effects of feed-forward central efferent activity, from afferent feedback from metabolites stimuli in the regulation of cardiovascular responses of exercise is post exercise circulatory occlusion (PECO) (Fisher et al. 2015). With PECO, a cuff rapidly inflated at the termination of exercise prevents arterial and venous blood flow, to effectively maintain the muscle in an exercised state with elevated metabolites, but without contractile activity or efferent commands. The effects of EIMD on cardiovascular responses to exercise and PECO remain unclear.

The aim of the present study was to investigate whether EIMD influences cardiovascular responses to isometric exercise and PECO. We hypothesized that EIMD would increase muscle afferent sensitivity and, accordingly, increase blood pressure responses to exercise and PECO.

## Methods

### Participants

The study received ethical approval from the Faculty of Science and Engineering Research Ethics and Governance Committee (reference number: 37464) and conformed to the Declaration of Helsinki. The inclusion criteria were males or females aged 18–31 years who were willing to abstain from caffeine and large meal consumption for 2 h prior to participation, as well as alcohol use and intense exercise for 2 days prior to any laboratory visits. Exclusion criteria included: use of non-steroidal anti-inflammatory medication (NSAIDS) and presence of injury or medical conditions that prevented resistance exercise participation. Twenty physically active, healthy volunteers (age 23.4 ± 4.0 years; mass 70.6 ± 13.4 kg; stature 1.73 ± 0.11 m; training 5.4 ± 3.3 h/week (mean ± SD)) were eligible and provided written, informed consent to take part in the study.

### Experimental design

**Fig. 1:**
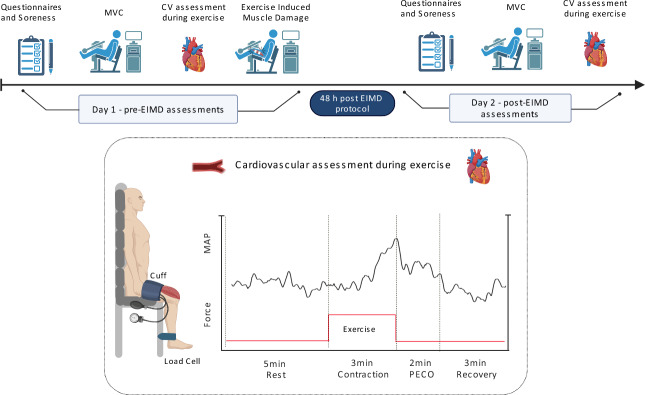
Experimental design and Cardiovascular assessment during exercise. Abbreviations: *MVC*  Maximal Voluntary contraction, *EIMD*  Exercise induced muscle damage, *MAP*  Mean arterial Pressure, *PECO*  Post-exercise circulatory occlusion, *CV* Cardiovascular. Created with BioRender.com

Participants visited the laboratory for a familiarization session, verbal explanation of the study procedures and to agree an appointment for the first experimental session (pre-EIMD) and the 48-h follow-up (post-EIMD). Assessments at pre-EIMD and post-EIMD followed the same procedures, with the exception that the EIMD exercise protocol was completed only once, which was at the end of the pre-EIMD session (Fig. 1).

### Questionnaires and soreness assessment

A physical activity readiness questionnaire (PAR-Q) was completed and standing stature and body mass were measured. Perceived muscle soreness of the knee extensors was measured using a visual analogue scale (VAS_SQ_) as participants held a single-leg squat on the dominant limb with the knee flexed at 90 ° (Burt et al. 2012). The assessment was made by asking participants to mark a “X” along a 10 cm scale to indicate the level of soreness: from 0 meaning no muscle soreness to 10 meaning muscles are too sore to move (Twist and Eston 2009; Burt et al. 2012).

### Maximal voluntary contraction assessment

Participants sat upright on a custom-made chair with hips and knees flexed at 90 ° and straps secured around the waist to minimise extraneous movements. Single leg maximal voluntary knee extension isometric contraction (MVC) was tested on the dominant limb with the leg secured 2 cm proximal to the malleolus by an inextensible strap connected at the other end to a calibrated load cell. Force signals were amplified and recorded (PowerLab 16/30; ML880, ADInstruments, Bellavista, NSW, Australia) and real-time feedback of force was available on a computer monitor. A warm-up was provided consisting of six isometric contractions at 50–80% maximal effort. After a 1 min rest, participants performed three MVCs, each separated by 1 min rest. The highest external force value was accepted as participant’s MVC (Mcphee et al. 2014).

### Cardiovascular assessment during exercise

Participants remained seated in the chair used for testing MVC and rested for 10 min, in which time the non-invasive blood pressure photoplethysmography (Human NIBP nano, ADI Instruments Systems, Oxford, UK) for continuous blood pressure monitoring and a 3-lead ECG were connected to PowerLab (16/30; ML880, ADInstruments, Bellavista, NSW, Australia). A rapid inflatable cuff (16 cm wide; E. Hokanson, Inc. Bellevue, WA 98005 USA) was positioned around the proximal thigh of the exercising leg and was inflated to 220–240 mmHg for 2 min to record BP responses at baseline. The cuff was then rapidly deflated, and participants rested for 10 min, or until BP returned to baseline values. Thereafter, participants performed a sustained isometric knee extension for 180 s at 30% of the MVC using the dominant limb. At the end of the 180 s contraction period, the cuff was rapidly inflated around the exercising thigh to 220–240 mmHg for post-exercise circulatory occlusion (PECO) and it remained inflated for 2 min. PECO prevents arterial and venous blood from entering/leaving the limb and therefore maintains the muscle metabolic environment in the exercised state. Participants rested for 3 min to recover at the end of PECO (Peçanha et al. 2021). Blood pressure monitoring was recorded throughout the duration of the entire protocol. Rate of perceived exertion (RPE) with Borg scale (Borg 1970) and self-reported level of pain (PAIN) with a numeric rating scale (NRS) (Karcioglu et al. 2018), were recorded at the end of every minute during the 3 min isometric sustained contraction.

### Exercise induced muscle damage protocol

The EIMD protocol consisted of unilateral eccentric knee extensor contractions on the dominant limb using a Kineo Multistation machine (Globus, Italy) that provided isokinetic mode and enabled the eccentric load to be accurately and rapidly adjusted in relation to the concentric load. A warm-up was provided consisting of 10 isokinetic concentric leg extensions performed through the full range of motion. The maximal concentric and eccentric torques were then assessed to enable the EIMD exercise to be set proportional to the maximal eccentric torque. Participants then completed repeated sets of 10 dynamic eccentric knee extensions in isotonic mode with the load set at 100% of the eccentric peak torque and participants were asked to give a maximal effort to oppose the load. The concentric load was set at 50% of concentric peak torque. The average speed was of 60 °/sec for concentric and eccentric loads, and full range of motion was set from 15 to 85 ° of knee flexion. At the end of each set of 10 repetitions, an MVC isometric force was tested, and exercise was terminated when MVC was reduced by 40% compared with starting values (Byrne et al. 2004; Szczyglowski et al. 2017).

### Data handling

Data collected through LabChart 8 software (ADInstruments, Bellavista, NSW, Australia) was imported into Microsoft Excel for calculation of the main variables. Cardiovascular outcomes were analysed for each given phase and presented as deltas (Δ-) calculated subtracting the average resting values from the average value (highest 60 s segments) of the different phases (contraction, PECO, and recovery) (Peçanha et al. 2021). All measurements were collected following the standards set up by the Task Force of the European Society of Cardiology and North American Society of Pacing and Electrophysiology (Force 1996). Stroke Volume (SV) and Cardiac Output (CO) were indirectly calculated from the Modelflow algorithm (Kenfack et al. 2004).

### Statistical analysis

The Shapiro–Wilk test was used to verify data distribution. Homogeneity of variance was verified by the Levene test, and sphericity by the Mauchly test. A paired t-test was performed for pre-EIMD and post-EIMD values for MVC, VAS_SQ_, cardiovascular responses at rest average pain and RPE during the 3 min sustained isometric contraction. Cardiovascular responses to exercise were assessed with a two-way repeated measures Analysis of Variance (ANOVA) for delta values of MAP, HR, CO, SV values using two within subject factors (Time: pre- and post-EIMD) and three phases (ΔContr; ΔPECO, and ΔRec). Pearson’s Product Moment Correlations were used to assess relationships between continuous variables. Single correlation analysis was performed for MVC, VAS_SQ_, Pain, RPE with Deltas for MAP responses pre- and post-EIMD, to investigate correlations between level of soreness, pain, and exertion with delta changes in MAP during contraction, PECO, and recovery.

## Results

Twenty participants (11 male; 9 female) aged 23.1 ± 3.9 years and body mass index 23.6 ± 2.4 kg/m^2^ (mean ± SD) took part in the study. All participants successfully completed the experimental procedures.

Isometric MVC was lower post-EIMD compared with pre-EIMD values (p < 0.001) and VAS_SQ_, RPE and Pain were significantly higher (p < 0.001) post-EIMD compared with pre-EIMD values. Data are reported in Table 1.

**Table 1:** Direct and Indirect measure of DOMS and cardiovascular responses at rest pre- and post-EIMD

Resting variables	Pre-EIMD	Post-EIMD
MVC (N)	634.6 ± 229.3	504.0 ± 160.5*
VAS_SQ_ (mm)	2.3 ± 1.5	39.5 ± 27.3*
MAP (mmhg)	98.3 ± 14.9	97.1 ± 12.5
Heart rate (bpm)	78.1 ± 10.7	75.9 ± 10.7
Cardiac output (l/min)	3.1 ± 0.6	2.8 ± 0.53
Stroke volume (ml)	39.6 ± 6.5	36.6 ± 5.3
RPE mean (6–20)	13.05 ± 1.82	14.7 ± 1.93*
Pain mean (0–10)	4.02 ± 1.79	5.90 ± 1.27*

Data are mean ± SD. Abbreviations: *MAP*  Mean arterial pressure, *VAS*_*SQ*_  Self-perceived soreness, *RPE*  Rate of perceived exertion. *p < 0.05 between pre- and post-EIMD

Rapid inflation of the thigh cuff over resting muscle had no significant effect on MAP either before EIMD (– 1.5 ± 5.4 mmHg, p = 0.243 mmHg) or 48 h after EIMD (– 1.6 ± 4.7 mmHg, p = 0.131). These responses before and after EIMD were not significantly different (p = 0.887).

**Fig. 2:**
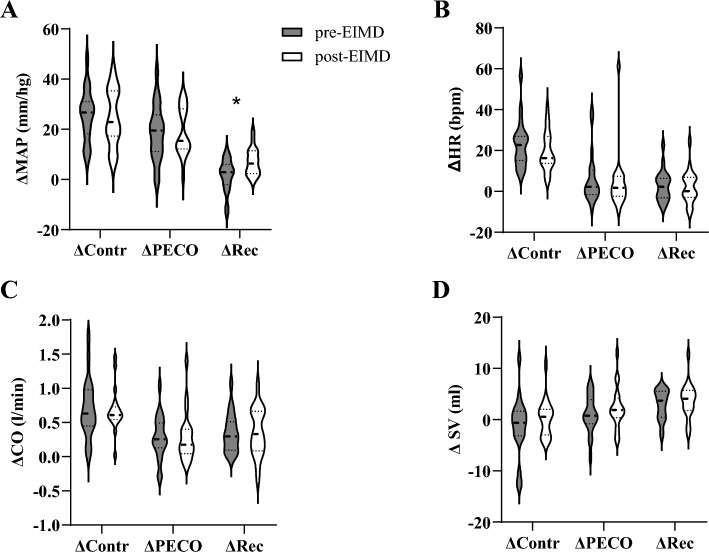
Changes in MAP and central Haemodynamics following EIMD. Panel** A** highlights changes in MAP while Panel** B**,** C** and** D** highlights changes in HR, CO, SV, respectively. Abbreviations: ΔContr = delta contraction; ΔPECO = delta post-exercise cuff occlusion; ΔRec = delta recovery, *MAP* Mean arterial Pressure, *HR* Heart Rate, *CO*  Cardiac Output, *SV*  Stroke Volume. Data are reported as mean ± SD. *p < 0.05 significant time × phase interaction

Indices of cardiovascular function were collected in the rested state pre-EIMD and post-EIMD and there were no significant differences between these time points (Table 1). Values for MAP, HR, SV and CO changed significantly through contraction to PECO and recovery phases (all p < 0.001; Fig. 2). There were no significant effects of time (pre- and post-EIMD) for any of the cardiovascular measurements during any of the phases (p = 0.354; p = 0.138; p = 0.952; p = 0.137). However, a significant time x phase interaction was found for MAP responses (p = 0.043, Fig. 2) as blood pressure was higher during the recovery phase post-EIMD compared with pre-EIMD values. ΔHR, SV and CO did not show significant time x phase interactions (p = 0.582; p = 0.887; p = 0.534).

**Fig. 3:**
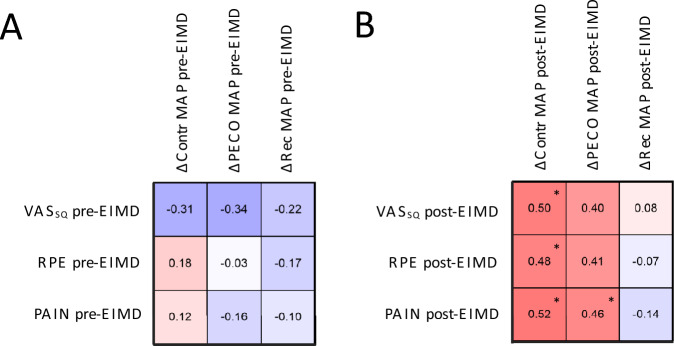
Heat map summarising associations between level of muscle soreness, perceived exertion, and pain with deltas in mean arterial pressure responses. Panel** A** highlights the associations at pre-EIMD while panel** B** highlights the associations at post-EIMD. Abbreviations: ΔContr MAP = difference between mean arterial blood pressure values at rest and contraction phases; ΔPECO = difference between mean arterial blood pressure values at rest and post-exercise cuff occlusion phases; ΔRec = difference between mean arterial blood pressure values at rest and recovery phases, *MAP*  Mean arterial pressure. VAS_SQ_ = soreness during squat exercise, *RPE*  Rate of perceived exertion, *PAIN*  self-reported perceived pain Data are presented as Pearson Coefficient (*r*). * = *p* < 0.05. Created with BioRender.com

Correlations analysis performed for VAS_SQ_, Pain and RPE with Deltas for MAP responses from rest to contraction, PECO and recovery showed no significant correlations pre-EIMD (all p > 0.05, Fig. 3A). However, there were positive correlations post-EIMD for delta MAP response to contraction and PECO (Fig. 3B). There was no significant correlation between changes in MVC following EIMD with changes in MAP during contraction, PECO or recovery (r = 0.01, p = 0.945; r = – 0.16, p = 0.115; r = 0.05, p = 0.752, respectively).

## Discussion

Our results showed no changes of mean cardiovascular responses to isometric exercise and PECO 48 h after EIMD compared with baseline measurements. However, considerable individual variability existed in the effects of EIMD on loss of force, soreness and perceptions of pain and effort during contraction, as well as the MAP responses to exercise and PECO. We found that muscle soreness measured with VAS_SQ_, RPE and perceptions of pain during contraction were positively correlated with ΔMAP during contraction and PECO 48 h after EIMD (Fig. 3B), but these relationships did not exist in the pre-EIMD condition (Fig. 3A). Similar to our findings, previous investigations reported no change in mean blood pressure responses after EIMD (Ray et al. 1998; Miles et al. 1997), but also found elevated self-perceived pain and RPE during muscle contractions, suggesting an increased afferent activity (Miles et al. 1997; Ray et al. 1998). Taken together, these findings support the hypothesis that muscle afferents are sensitised in individuals reporting more severe effects of EIMD and accordingly, the blood pressure responses to contraction and PECO are increased for those individuals.

Sensitisation likely affects both metabo- and mechano- sensitive muscle nerve afferents, as well as nociceptors (Ota et al. 2013; Fujii et al. 2008; Matsubara et al. 2019). Metaboreceptors are activated by metabolites produced during muscle contractions (Light et al. 2008; MacLean et al. 2000) generating increases in sympathetic nerve activity and regulating cardiovascular responses during exercise (Boushel 2010). PECO has been largely used to investigate effects of metaboreflex on cardiovascular responses to exercise, without the confounding effects of central command and mechanoreflex activity (Fisher et al. 2015). In brief, metabolites trapped inside the muscle during PECO continue to stimulate metabo-sensitive muscle nerve afferents, thereby sustaining the metaboreflex cardiovascular responses to exercise (Alam and Smirk 1937). PECO has often been used to study metaboreflex activation, but several studies also reported an increase in BP following mechanical compression (Paintal 1960; Ge and Khalsa 2003). It is possible, therefore, that external compression applied over the muscles activated mechanoreceptors during PECO. In the present study, inflating the thigh cuff over rested muscle did not change MAP, either in the pre- or the post-EIMD conditions, suggesting that the mechanoreflex alone was not responsible for the MAP changes. However, EIMD-induced swelling of the damaged muscle can increase mechanoreceptor sensitivity due to raised interstitial pressures (Ray et al. 1998) which may have increased mechanoreceptor sensitivity during and after the sustained contraction. Previous research showed a mechanical sensitisation during mechanoreceptors activation, when metabolites accumulated (Bell and White 2005; Carrington et al. 1985), including ATP, cyclooxygenase, bradykinin, lactate, and H^+^ (Li and Sinoway 2002; Middlekauff and Chiu 2004; Cui et al. 2008; Drew et al. 2008; Rotto et al. 1985). Studies of rat muscles also showed that lowering pH to levels seen during muscle contractions can sensitise metaboreceptors to mechanical stimulus (Hotta et al. 2010). It is therefore possible that mechanoreceptors increased their sensitivity during the sustained muscle contractions and contributed together with metaboreceptors to the MAP changes we observed following EIMD.

In the present study, the intensity of the exercise contraction was moderate (13–15 RPE; 4–6 Pain). However, the accumulation of metabolites may have been relatively higher for those participants reporting more severe EIMD which could explain MAP increases during contraction and PECO were greater for those participants. Different subgroups of metaboreceptors are activated at different concentrations of metabolites, with some showing increased metabo-nociceptors activation only at higher metabolite concentrations (Jankowski et al. 2013; Light et al. 2008). The effects of specific metabolites on the cardiovascular response to exercise has been explored by past several studies. For instance, muscle deprotonated phosphate (H_2_PO_4_) (Sinoway et al. 1994), Pi, and pH (Boushel et al. 1998) were linked to BP increases during exercise. Additionally, Boushel et al. reported that pH or Pi remained close to the exercise values following cuff inflation during PECO, and correlated with MAP increases (Boushel et al. 1998). However, these findings occurred in a non-fatigued, healthy muscle and the linear correlations may therefore be different following EIMD where muscle metabolite concentrations may be different from the non-damaged condition. Indeed, Fouré et al. found an increased Pi concentration at rest and reduced rate of Pi production during exercise following EIMD (Fouré et al. 2015). Moreover, the authors reported reduced proton production (increased pH), hypothesising that processes in ATP production from oxidative and anaerobic pathways were impaired (Fouré et al. 2015). Furthermore, Davies et al. found no changes in the rate of Pi, Pi/PCr or pH differences during an incremental exercise task 48 h post-EIMD (Davies et al. 1985). Thus, it remains unclear which metabolite changes sensitise muscle afferents with EIMD to increase MAP responses to exercise and PECO.

An important limitation to the present work is that we did not measure metabolites accumulation during exercise and PECO. Measures such as nuclear magnetic resonance spectroscopy or collection of muscle biopsies in future studies could reveal the changes in muscle metabolism during exercise and PECO, both before and after EIMD that influence muscle afferent nerve activity and MAP responses.

## Conclusion

We found that muscle soreness, RPE and perception of pain during muscle contractions and PECO were positively correlated with changes in MAP during 3-min sustained isometric exercise and PECO after EIMD. This suggests that muscle afferents are sensitised in individuals reporting more severe effects of EIMD, which causes greater blood pressure responses for those individuals.

## Data Availability

Data available upon reasonable request from the authors.

## Abbreviations

ANOVA: Analysis of variance
ATP: Adenosine triphosphate
BMI: Body mass index
BP: Blood pressure
CO: Cardiac output
CV: Cardiovascular
EIMD: Exercise-induced muscle damage
EPR: Exercise pressor reflex
HR: Heart rate
H^+^: Hydrogen Ion
MVC: Maximal voluntary contraction
MAP: Mean arterial pressure
PAR-Q: A physical activity readiness questionnaire
PCr: Creatin-phosphate
Pi: Deprotonated phosphate
PECO: Post-exercise circulatory occlusion
RPE: Rate of perceived exertion
SV: Stroke volume
VAS: Visual analog scale
